# Central nervous system manifestations in COVID‐19 patients: A systematic review and meta‐analysis

**DOI:** 10.1002/brb3.2025

**Published:** 2021-01-09

**Authors:** Shahrzad Nazari, Amirhossein Azari Jafari, Seyyedmohammadsadeq Mirmoeeni, Saeid Sadeghian, Mohammad Eghbal Heidari, Siavash Sadeghian, Farhad Assarzadegan, Seyed Mahmoud Puormand, Hamid Ebadi, Davood Fathi, Sahar Dalvand

**Affiliations:** ^1^ Department of Neuroscience and Addiction Studies School of Advanced Technologies in Medicine Tehran University of Medical Sciences Tehran Iran; ^2^ Student Research Committee School of Medicine Shahroud University of Medical Sciences Shahroud Iran; ^3^ Department of Paediatric Neurology Golestan Medical, Educational, and Research Centre Ahvaz Jundishapur University of Medical Sciences Ahvaz Iran; ^4^ Students Scientific Research Center Tehran University Medical Science Tehran Iran; ^5^ Ahvaz Jundishapur University of Medical Sciences Ahvaz Iran; ^6^ Department of Neurology, Imam Hossein Hospital Shahid Beheshti University of Medical Sciences Tehran Iran; ^7^ Imam Hossein Hospital Shahid Beheshti University of Medical Sciences Tehran Iran; ^8^ Department of Clinical Neurosciences University of Calgary Calgary AB Canada; ^9^ Brain and Spinal Cord Injury Research Center, Neuroscience Institute Tehran University of Medical Sciences Tehran Iran; ^10^ Department of Neurology, Shariati Hospital Tehran University of Medical Sciences Tehran Iran; ^11^ Functional Neurosurgery Research Center, Shohada Tajrish Comprehensive Neurosurgical Center of Excellence Shahid Beheshti University of Medical Sciences Tehran Iran

**Keywords:** consciousness disorders, COVID‐19, dizziness, headache, nervous system diseases, SARS‐CoV‐2 infection

## Abstract

**Background:**

At the end of December 2019, a novel respiratory infection, initially reported in China, known as COVID‐19 initially reported in China, and later known as COVID‐19, led to a global pandemic. Despite many studies reporting respiratory infections as the primary manifestations of this illness, an increasing number of investigations have focused on the central nervous system (CNS) manifestations in COVID‐19. In this study, we aimed to evaluate the CNS presentations in COVID‐19 patients in an attempt to identify the common CNS features and provide a better overview to tackle this new pandemic.

**Methods:**

In this systematic review and meta‐analysis, we searched PubMed, Web of Science, Ovid, EMBASE, Scopus, and Google Scholar. Included studies were publications that reported the CNS features between 1 January 2020 and 20 April 2020. The data of selected studies were screened and extracted independently by four reviewers. Extracted data analyzed by using STATA statistical software. The study protocol registered with PROSPERO (CRD42020184456).

**Results:**

Of 2,353 retrieved studies, we selected 64 studies with 11,687 patients after screening. Most of the studies were conducted in China (58 studies). The most common CNS symptom of COVID‐19 was headache (8.69%, 95%CI: 6.76%–10.82%), dizziness (5.94%, 95%CI: 3.66%–8.22%), and impaired consciousness (1.90%, 95%CI: 1.0%–2.79%).

**Conclusions:**

The growing number of studies has reported COVID‐19, CNS presentations as remarkable manifestations that happen. Hence, understanding the CNS characteristics of COVID‐19 can help us for better diagnosis and ultimately prevention of worse outcomes.

## Graphical abstract

The most common CNS symptom of COVID‐19 was headache (8.69%, 95%CI: 6.76%–10.82%), dizziness (5.94%, 95%CI: 3.66%–8.22%), and impaired consciousness (1.90%, 95%CI: 1.0%–2.79%). The growing number of studies has reported COVID‐19, CNS presentations as remarkable manifestations that happen. Hence, understanding the CNS characteristics of COVID‐19 can help us for better diagnosis and ultimately prevention of worse outcomes.

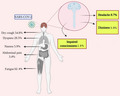

## 1 INTRODUCTION

At the end of December 2019, a novel respiratory syndrome, known as COVID‐19, was reported in Wuhan city, Hubei province, China. The first sign of this infection (2019‐nCoV, COVID‐19) was pneumonia (Adhikari et al., 2020; WHO, 2020; Shi, Qin, et al., 2020; Velavan & Meyer, 2020; Wang, Hu, et al., 2020; Wang, Wang, et al., 2020; Wu, Chen, et al., 2020). This new pandemic rapidly spread worldwide, and an increasing number of infected cases and deaths have been reported globally (Jiang et al., 2020; Sohrabi et al., 2020). Hence, the COVID‐19 outbreak was officially considered as a Public Health Emergency of International Concern (PHEIC) by the World Health Organization (WHO) Emergency Committee (Mackenzie & Smith, 2020; WHO, 2020). Severe acute respiratory syndrome coronavirus 2 (SARS‐CoV‐2) is a zoonotic pathogen and can transmit from infected animals (such as bats and snakes) to humans eventually leading to epidemics and pandemics through human‐to‐human transmission (Hassan et al., 2020; Mackenzie & Smith, 2020). Most cases of COVID‐19 have shown respiratory symptoms ranging from cough to dyspnea and respiratory failure as well as the typical signs and symptoms of infection such as fever and fatigue (Cascella et al., 2020; Chen, Zhou, et al., 2020; Wang, Hu, et al., 2020; Young et al., 2020).

However, a growing number of COVID‐19 patients are presenting with different combinations of the central nervous system (CNS) manifestations (Asadi‐Pooya & Simani, 2020; Mao et al., 2020; Montalvan et al., 2020). Several case reports have indicated the presence of various CNS complications, including encephalitis, stroke, meningitis, and encephalopathy in COVID‐19 patients (Co et al., 2020; Filatov et al., 2020; Moriguchi et al., 2020; Zhou, Zhang, et al., 2020). Furthermore, a large observational study carried out by Mao et al. shows the prevalence of the CNS presentations such as dizziness, headache, impaired consciousness, acute cerebrovascular disease, ataxia, and seizure (Mao et al., 2020). Therefore, awareness of the different aspects of the short‐ and long‐term effects of this virus on the central nervous system could decently guide scientists. In this systematic review and meta‐analysis, we assessed the CNS manifestations in COVID‐19 cases.

## 2 METHOD

### 2.1 Search strategy and selection criteria

We performed this systematic review and meta‐analysis based on Preferred Reporting Items for Systematic Reviews and Meta‐Analyses (PRISMA) guidelines (Moher et al., 2009), and our study protocol is submitted to PROSPERO (ID: CRD42020184456). We systematically searched six databases including Google Scholar, Scopus, PubMed, Web of science, Ovid, and EMBASE for all published articles from 1 January 2020 until 20 April 2020 using the following Medical Subject Heading terms (MESH terms):

(“Wuhan coronavirus” OR “Wuhan seafood market pneumonia virus” OR “COVID19 virus” OR “COVID‐19 virus” OR “coronavirus disease 2019 virus” OR “SARS‐CoV‐2” OR “SARS2” OR “2019‐nCoV” OR “2019 novel coronavirus” OR “2019‐nCoV infection” OR “2019 novel coronavirus disease” OR “2019‐nCoV disease” OR “coronavirus disease‐19” OR “coronavirus disease 2019” OR “2019 novel coronavirus infection” OR “COVID19” OR “COVID‐19” OR “severe acute respiratory syndrome coronavirus 2” OR “coronavirus*”) AND (“Manifestation, Neurologic” OR “Neurological Manifestations” OR “Neurologic Manifestation” OR “Neurological Manifestation” OR “Neurologic Symptom” OR “CNS” OR “brain” OR “neuro*” OR “headache” OR “dizziness” OR “ataxia” OR “epilepsy” OR “seizure” OR “migraine*” OR “CSF” OR “Cerebrospinal Fluids” OR “Fluid, Cerebrospinal” OR “Fluids, Cerebrospinal” OR “Cerebro Spinal Fluid” OR “Cerebro Spinal Fluids” OR “Fluid, Cerebro Spinal” OR “Fluids, Cerebro Spinal” OR “Spinal Fluid, Cerebro” OR “Spinal Fluids, Cerebro” OR “stroke” OR “vertigo” OR “consciousness” OR “Impaired consciousness” OR “coma” OR “cerebrovascular disease” OR “acute cerebrovascular disease” OR “encephalitis”) alone or in combination with OR and AND operators.

After removing the duplicated records, articles were screened based on their titles and abstracts by two authors (S.S and M.H) independently. The full texts of eligible publications were examined for inclusion and exclusion criteria (A.AJ, S.M, S.S, and M.H). Observational studies reported at least one of the related CNS symptoms in COVID‐19 patients without any language, race, country, and gender limitations included for quantitative synthesis. The preprint studies, interventional studies, systematic reviews, case reports, conferences, commentaries, letters, editorial, author responses, correspondence articles, in vitro, animal studies, children population, articles without full text, or unreliable data were excluded. In addition, the reference list of the eligible studies was searched to prevent missing publication and include all related literature. The data were independently extracted (A.AJ. S.M, S.S, S.S, and M.H), and discrepancies were resolved with discussion and consensus by three independent researchers (SH.N, S.D, and F.A).

### 2.2 Data analysis and quality assessment

The desired data were recorded using an excel spreadsheet form that included the title, first author, year and month of publication, type of study, country, total sample size, the sample size of male and female, study design, demographic characteristics, exposure history, clinical manifestation, CNS symptoms, and any reported comorbidity.

We assessed the quality of included studies (A.AJ. S.M, S.S, S.S, and M.H), based on the NIH quality assessment tool for observational cohort and case series studies (NIH). This instrument assessed the quality of included studies based on the research question, study population, the participation rate of eligible persons, inclusion and exclusion criteria, sample size justification, analyses, reasonable timeframe, exposure, outcome measures, outcome assessors, and loss to follow‐up.

### 2.3 Meta‐analysis

Data from included studies were extracted for the number of events and total patients to perform a meta‐analysis (S.D). Cochrane's *Q* test and the *I* ^2^ index were used to assess heterogeneity among selected studies. Heterogeneity was categorized as low (below 25%), moderate (25%–75%), and high (above 75%) (Higgins & Thompson, 2002). Also, data adjusted by Freeman–Tukey double arcsine transformation and their 95% CIs were calculated by the Clopper–Pearson method (Clopper & Pearson, 1934). We calculate mean and standard deviations from median and quartiles by using Wan method (Wan et al., 2014). For continuous data, we estimate pooled results of means and their respective 95% CI by the inverse variance method. All analyses were performed using STATA statistical software, version 13 (StataCorp).

## 3 RESULTS

**FIGURE 1:**
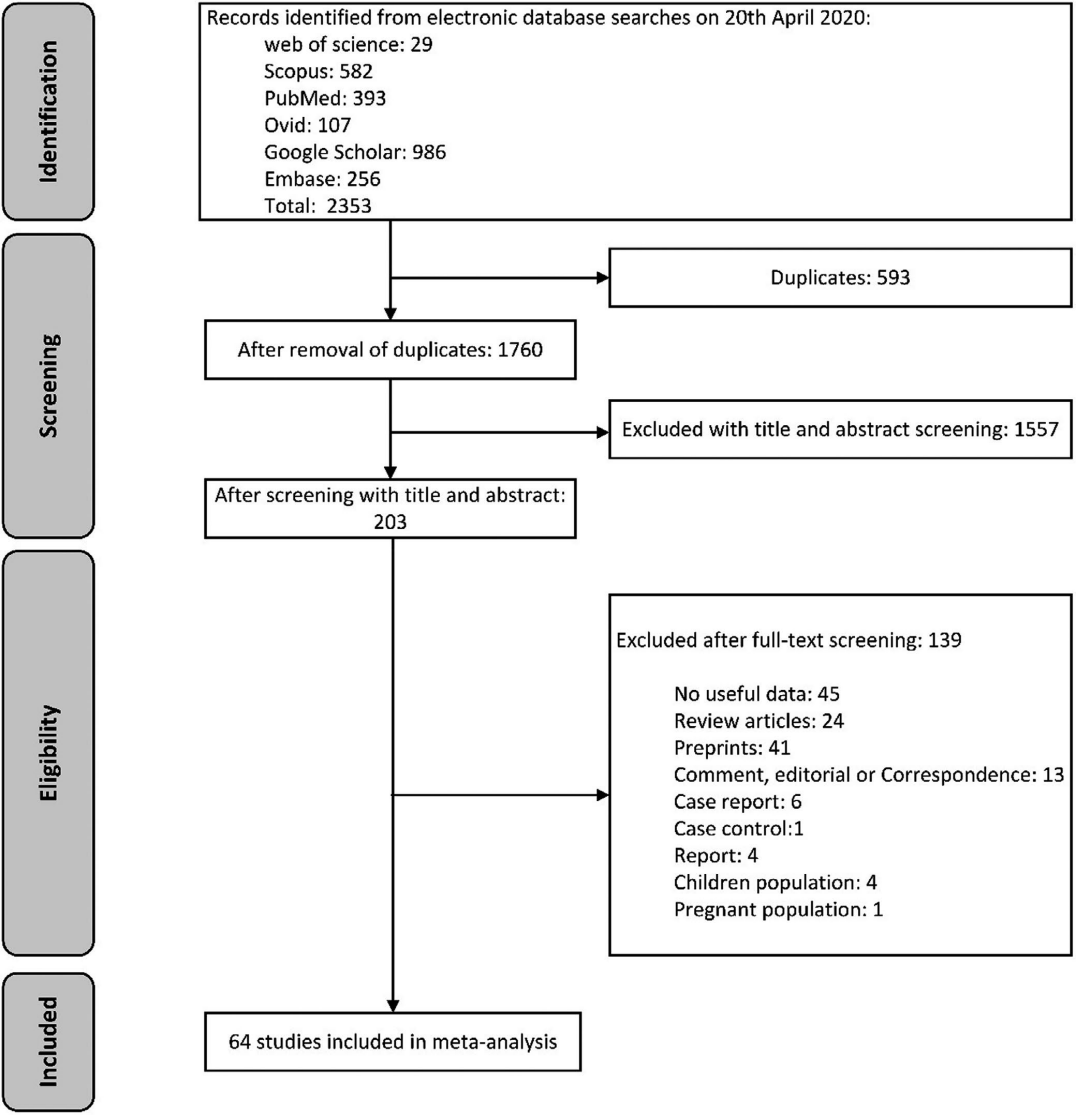
The process of surveying, screening, and selecting the articles for this systematic review and meta‐analysis based on PRISMA guideline

As illustrated in (Figure 1), a total of 2,353 studies were retrieved after a systematic search in the aforementioned databases. After removing duplicates, 1,760 studies remained. Then, we narrowed the studies to 203 articles by screening with titles and abstracts. In full‐text screening, 45 studies with no reliable or useful data, 24 review articles, 41 preprints, 6 case reports, 1 case controls, 4 reports, 4 papers with specific children population, one study with specific pregnant population, and 13 publications such as Commentary, editorial or Correspondence letters were excluded. Finally, 64 studies (Barrasa et al., 2020; Chen, Chen, et al., 2020; Chen, Qi, et al., 2020; Chen, Wu, et al., 2020; Chen, Yan, et al., 2020; Chen, Yang, et al., 2020; Chen, Zhou, et al., 2020; Cheng et al., 2020; Ding et al., 2020; Du, Liu, et al., 2020; Du, Tu, et al., 2020; Feng et al., 2020; Guan et al., 2020; Guo et al., 2020; Gupta et al., 2020; Han et al., 2020; Hsih et al., 2020; Huang et al., 2020; Jia et al., 2020; Jin et al., 2020; Kim et al., 2020; Kong et al., 2020; Lei, Huang, et al., 2020; Lei, Jiang, et al., 2020b; Li, Wang, et al., 2020; Liang et al., 2020; Ling et al., 2020; Liu, Yang, et al., 2020; Liu, Yang, et al., 2020; Liu, Yang, et al., 2020; Lo et al., 2020; Mao et al., 2020; Mi et al., 2020; Mo et al., 2020; Moein et al., 2020; Peng, Liu, et al., 2020; Peng, Meng, et al., 2020; Qian et al., 2020; Qin et al., 2020; Shao et al., 2020; Shi, Han, et al., 2020; Shi, Qin, et al., 2020; Song et al., 2020; Sun et al., 2020; Tan et al., 2020; Tian et al., 2020; Wan, Xiang, et al., 2020; Wan, Yi, et al., 2020; Wang, Hu, et al., 2020; Wang, He, et al., 2020; Wang, Fang, et al., 2020; Wu, Chen, et al., 2020; Wu, Wu, et al., 2020; Xu et al., 2020; Yang, Cao, et al., 2020; Yang, Yu, et al., 2020; Yu et al., 2020; Zhang, Cai, et al., 2020; Zhang, Wang, et al., 2020; Zhao et al., 2020; Zheng, Tang, et al., 2020; Zheng, Xu, et al., 2020; Zhong et al., 2020; Zhu et al., 2020) including 11,282 COVID‐19 patients, met our inclusion criteria, and were entered in meta‐analysis. The main characteristics of our included studies are presented in Table 1.

**TABLE 1:** Demographics and baseline characteristics of included studies with COVID‐19‐infected patients presenting CNS symptoms

Author	Month of publication	Type of studies	Country, City/Province	Sample size	No of positive cases (female/male)	Quality assessment
Yang, Yu, et al. (2020)	February 2020	Cohort	China, Wuhan	52	17/35	Good
Yang, Cao, et al. (2020)	February 2020	Cohort	China, Wenzhou	149	68/81	Fair
Wang, Hu, et al. (2020)	March 2020	Case series	China, Wuhan	138	63/75	Good
Song et al. (2020)	February 2020	Retrospective	China, –	51	26/25	Good
Shi, Han, et al. (2020)	March 2020	Cohort	China, Wuhan	81	39/42	Good
Qian et al. (2020)	March 2020	Case series	China, Zhejiang province	91	54/37	Good
Mao et al. (2020)	April 2020	Case series	China, Wuhan	214	127/87	Good
Liu, Yang, et al. (2020)	February 2020	Case series	China, –	12	4/8	Fair
Liu, Yang, et al. (2020)	February 2020	Retrospective	China, Wuhan	137	76/61	Fair
Li, Fang, et al. (2020)	March 2020	Cross‐Sectional	China, ‐	54	32/22	Fair
Du, Tu, et al. (2020)	April 2020	Cohort	China, Wuhan	85	23/62	Fair
Cheng et al. (2020)	March 2020	Cross‐Sectional	China,	1,079	505/573	Fair
Chen, Zhou, et al. (2020)	January 2020	Retrospective	China, Wuhan	99	32/67	Good
Zhu et al. (2020)	March 2020	Retrospective	China, Anhui province	32	17/15	Good
Wu, Chen, et al. (2020)	March 2020	Cohort	China, Wuhan	191	72/119	Good
Zhong et al. (2020)	March 2020	Cohort	China, Wuhan	49	42/7	Fair
Zheng, Xu, et al. (2020)	April 2020	Case series	China, Changsha	99	48/51	Fair
Zheng, Tang, et al. (2020)	March 2020	Retrospective	China, Changsha	161	81/80	Good
Zhao et al. (2020)	March 2020	Retrospective Cohort	China, Changsha	118	58/60	Good
Zhang, Cai, et al. (2020)	March 2020	Retrospective	China, –	573	278/295	Good
Zhang, Wang, et al. (2020)	April 2020	Retrospective Cohort	China, Wuhan	663	342/321	Good
Yu et al. (2020)	March 2020	Prospective Cohort	China, Beijing	76	38/38	Fair
Xu et al. (2020)	February 2020	Retrospective	China, Beijing	50	21/29	Fair
Wu, Wu, et al. (2020)	February 2020	Cross‐Sectional	China, Chongqing	80	38/42	Fair
Wang, Hu, et al. (2020)	March 2020	Retrospective	China, Wuhan	1,012	488/524	Good
Wang, He, et al. (2020)	March 2020	Retrospective study	China, Wuhan	339	173/166	Good
Wan, Yi, et al. (2020)	March 2020	Cross sectional	China, Chongqing	123	57/66	Good
Wan, Xiang, et al. (2020)	2020	Case series	China, Chongqing	135	63/72	Good
Tian et al. (2020)	February 2020	Retrospective observational	China, Beijing	262	135/127	Fair
Tan et al. (2020)	April 2020	Retrospective observational	China, Changsha	27	16/11	Fair
Sun et al. (2020)	April 2020	Retrospective observational	China, Nanyang	150	83/67	Good
Shi, Qin, et al. (2020)	March 2020	Retrospective observational	China, Wuhan	416	211/205	Fair
Shao et al. (2020)	April 2020	Retrospective observational	China, Wuhan	136	46/90	Good
Qin et al. (2020)	March 2020	Retrospective observational	China, Wuhan	452	217/235	Good
Peng, Meng, et al. (2020)	March 2020	Retrospective observational	China, Wuhan	112	59/53	Fair
Peng, Liu, et al. (2020)	April 2020	Cross‐sectional	China, Shanghai	86	47/39	Fair
Moein et al. (2020)	2020	Retrospective observational	Iran, Tehran	60	20/40	Fair
Mo et al. (2020)	2020	Retrospective	China, Wuhan	155	69/86	Good
Mi et al. (2020)	2020	Retrospective	China, Wuhan	10	8/2	Fair
Lo et al. (2020)	March 2020	Retrospective	China, Macau	10	7/3	Fair
Liu, He, et al. (2020)	February 2020	Retrospective	China, Wuhan	30	20/10	Fair
Ling et al. (2020)	2020	Retrospective	China, Wuhan	8	4/4	Poor
Liang et al. (2020)	March 2020	Retrospective	China, Wuhan	88	37/51	Good
Lei, Jiang, et al. (2020)	2020	Retrospective	China, Wuhan	34	20/14	Good
Lei, Huang, et al. (2020)	2020	Retrospective	China, Guiyang	14	6/8	Good
Kong et al. (2020)	February 2020	Case series	South Korea, National survey	28	13/15	Poor
Kim et al. (2020)	2020	Retrospective	Korea, National survey	28	13/15	Good
Jin et al. (2020)	March 2020	Retrospective	China, Zhejiang	651	320/331	Good
Jia et al. (2020)	2020	Retrospective	China, Qingdao	44	29/15	Fair
Huang et al. (2020)	January 2020	Retrospective	China, Wuhan	41	11/30	Good
Hsih et al. (2020)	2020	Retrospective	Taiwan, Taichung	43	26/13	Fair
Han et al. (2020)	2020	Retrospective	China, Wuhan	108	70/38	Fair
Gupta et al. (2020)	April 2020	Case series	India, New Delhi	21	7/14	Fair
Guo et al. (2020)	2020	Retrospective	China, Wuhan	174	98/76	Good
Guan et al. (2020)	February 2020	cross	China, National	1,099	459/640	Good
Feng et al. (2020)	April 2020	Retrospective	China, Wuhan, Shanghai and Anhui	476	205/271	Good
Du, Liu, et al. (2020)	April 2020	Retrospective	China, Wuhan	109	35/74	Good
Ding et al. (2020)	March 2020	Case series	China, Wuhan	5	3/2	Good
Chen, Chen, et al. (2020)	April 2020	Retrospective	China, Wuhan	42	27/15	Fair
Chen, Yang, et al. (2020)	April 2020	Retrospective	China, ‐	104	52/52	Good
Chen, Wu, et al. (2020)	March 2020	Case series	China, Wuhan	274	103/171	Good
Chen, Qi, et al. (2020)	March 2020	Retrospective	China, Shanghai	249	123/126	Good
Chen, Yan, et al. (2020)	March 2020	Retrospective	China, Wuhan	150	66/84	Good
Barrasa et al. (2020)	2020	Case series	Spain, Vitoria	48	21/27	Fair

The total sample size of eligible studies was 11,687, including 5,568 females and 6,114 males. The mean age for noncritical patients was 48.557 (95% CI: 44.816%–52.299%) and for critical patients was 58.965 (95% CI: 55.792%–62.139%). As shown in Table 2, the proportion of patients with travel history to Wuhan, Wuhan‐related exposure, and Living in Wuhan was 51.15%, 78.52%, and 47.46%, respectively. In addition, the proportion of patients with travel history to other infected areas and contact with patients was 52.21% and 34.65%, respectively. Mortality was assessed in 25 studies with a pooled incidence rate of 10.47%. The incidence rate of positive females and males was 46.42% (95% CI: 43.01%–49.83%) and 49.50% (95% CI: 45.70%–53.31%), respectively. 36.17% (95% CI: 27.91%–44.84%) of infected patients were in the severe, critical, or intensive care unit condition. In addition, the incidence rate of mortality and survival was 10.47% (95% CI: 5.08%–17.33%) and 81.43% (95% CI: 65.75%–93.29%), respectively.

**TABLE 2:** Positive PCR, severity, mortality, and exposure history of COVID‐19‐infected patients having CNS symptoms

Variables	No of studies	Total sample size	No positive case	Incidence rate (95% CI)	Heterogeneity		
					*I* ^2^ (%)	*Q*	*p*‐Value
Positive female	60	11,425	5,363	0.4642 (0.4301–0.4983)	92.2	752.6	<.0001
Positive male	60	11,425	5,919	0.4950 (0.4570–0.5331)	93.8	957.8	<.0001
Severe or critical or ICU	40	9,821	2,611	0.3617 (0.2791–0.4484)	98.6	2,827.4	<.0001
Nonsevere or Noncritical or Non‐ICU	37	8,095	5,694	0.7061 (0.6229–0.7832)	98.3	2,154.0	<.0001
Mortality	25	7,087	556	0.1047 (0.0508–0.1733)	98.4	1,497.6	<.0001
Survival	18	3,174	2,585	0.8143 (0.6575–0.9329)	98.9	1,600.9	<.0001
Exposure history							
Travel history to Wuhan	27	6,476	3,434	0.5115 (0.3295–0.6920)	99.5	5,434.9	<.0001
Wuhan‐related exposure	2	567	433	0.7852 (0.7501–0.8183)	–	–	–
Living in Wuhan	2	1,151	535	0.4746 (0.4455–0.5037)	–	–	–
Travel history to other infected areas	3	255	133	0.5221 (0.4609–0.5832)	0.0	1.1	.5664
Contact with patients	19	4,422	1504	0.3465 (0.2976–0.3953)	90.1	181.2	<.0001
Family clustering	6	1,182	254	0.2044 (0.1376–0.2712)	84.9	33.1	<.0001
Unknown exposure history	6	472	63	0.1244 (0.0446–0.2042)	91.3	57.7	<.0001

**FIGURE 2:**
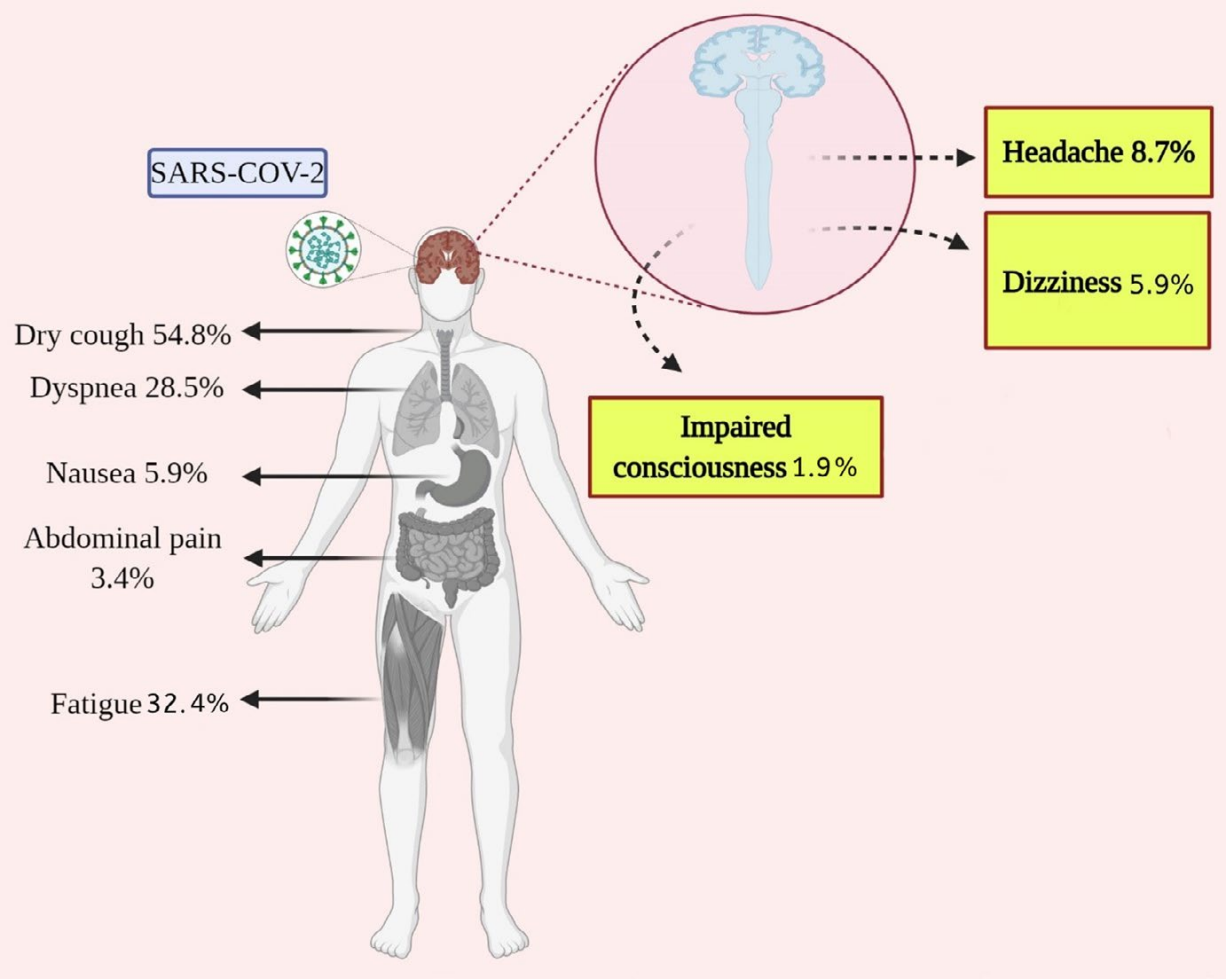
The incidence rate of CNS manifestations in COVID‐19 patients (this figure is Created with BioRender.com)

Based on the results shown in Table 3 and Figure 2, the most common manifestations were fever 79.39% (95% CI: 73.94%–84.37%), cough 54.77% (95%CI: 49.10%–60.38%), fatigue 32.39% (95% CI: 26.78%–38.0%), dyspnea 28.47% (95% CI: 21.49%–35.99%), chest tightness 23.83% (95% CI: 17.84%–29.82%), and shortness of breath 20.42% (95% CI: 13.28%–28.85%). The highest incidence rate among CNS symptoms of COVID‐19 patients was for headache (8.69% with 95% CI: 6.76%–10.82%), followed by dizziness (5.94%, 95%CI: 3.66%–8.22%), and impaired consciousness (1.90% with 95% CI: 1.0%–2.79%).

**TABLE 3:** Clinical Manifestations in COVID‐19‐infected patients presenting CNS symptoms

Variables	No of studies	Total sample size	No of positive case	Incidence rate (95% CI)	Heterogeneity		
					*I* ^2^ (%)	*Q*	*p*‐Value
General symptoms							
Fever	63	11,537	8,723	0.7939 (0.7394–0.8437)	97.7	2,689.5	<.0001
Fatigue	43	8,638	2,454	0.3239 (0.2678–0.3800)	97.8	1936.5	<.0001
Myalgia (muscle pain or muscle injury)	41	7,479	246	0.1395 (0.1169–0.1621)	88.2	338.4	<.0001
Nasal congestion	3	2,684	151	0.0554 (0.0428–0.0680)	51.2	4.1	.1290
Rhinorrhea	15	3,881	163	0.0447 (0.0258–0.0676)	83.9	87.2	<.0001
Dry cough or cough	62	11,507	6,047	0.5477 (0.4910–0.6038)	97.0	2054.7	<.0001
Arthralgia	3	204	8	0.0243 (0.000–0.0785)	63.6	5.5	.0642
Chill	11	3,878	512	0.1802 (0.0834–0.3021)	98.4	637.1	<.0001
GI symptoms	6	1,795	83	0.0501 (0.0148–0.0854)	87.7	40.8	<.0001
Nausea	11	1,934	115	0.0595 (0.0387–0.0803)	69.0	32.3	.0004
Vomiting	11	2,703	97	0.0322 (0.0255–0.0397)	23.6	13.1	.2183
Nausea and/or vomiting	13	3,160	181	0.0518 (0.0337–0.0700)	79.7	59.2	<.0001
Anorexia or inappetence	17	2,638	588	0.2052 (0.1393–0.2711)	96.8	508.7	<.0001
Diarrhea	45	8,270	909	0.1030 (0.0832–0.1227)	91.0	489.8	<.0001
Abdominal pain	14	3,132	112	0.0345 (0.0205–0.0485)	74.7	51.5	<.0001
Chest tightness	9	1,857	468	0.2383 (0.1784–0.2982)	88.3	68.6	<.0001
Shortness of breath	26	6,538	1,177	0.2042 (0.1328–0.2858)	98.1	1,329.0	<.0001
Dyspnea	32	4,793	1,255	0.2847 (0.2149–0.3599)	96.4	859.7	<.0001
Chest pain	13	2,490	68	0.0249 (0.0075–0.0490)	81.4	64.4	<.0001
Hemoptysis	13	3,518	71	0.0169 (0.0074–0.0289)	65.2	34.5	.0006
Heart palpitations	2	191	13	0.0671 (0.0316–0.1026)	–	–	–
Pharyngodynia or Throat pain or Pharyngalgia or throat sore	41	9,021	888	0.0983 (0.0767–0.1219)	89.9	399.9	<.0001
Coryza or sputum production or expectoration	30	7,239	1,909	0.2517 (0.1852–0.3182)	98.4	1791.4	<.0001
CNS symptoms							
Headache	48	9,782	897	0.0869 (0.0676–0.1082)	89.5	449.5	<.0001
Dizziness	10	2,296	139	0.0594 (0.0366–0.0822)	81.3	48.1	<.0001
Headache and/or Dizziness	5	558	58	0.0978 (0.0733–0.1224)	6.2	4.3	.3711
Impaired consciousness	2	877	26	0.0190 (0.0100–0.0279)	–	–	–

Table 4 shows comorbidities that were reported in 60 studies. The highest incidence rate in comorbidities was hypertension with 23.54% (95% CI: 19.14%–27.94%), diabetes mellitus (11.68% with 95% CI, 9.80%–13.57%), cardiovascular disease (11.66% with 95% CI: 8.97%–14.35%), and cerebrovascular diseases (3.47% with 95% CI: 2.29%–4.85%).

**TABLE 4:** Comorbidities in COVID‐19‐infected patients with CNS symptoms

Variables	No of studies	Total sample size	NO positive case	Incidence rate (95% CI)	Heterogeneity		
					*I* ^2^ (%)	*Q*	*p*‐Value
Any comorbidities	27	6,729	1,932	0.3247 (0.2729–0.3766)	95.8	617.9	<.0001
Cerebrovascular diseases	19	4,502	152	0.0347 (0.0229–0.0485)	76.3	76.1	<.0001
Cardiovascular diseases	35	8,394	743	0.1166 (0.0897–0.1435)	97.8	1577.8	<.0001
Cardiovascular disease and Cerebrovascular diseases	8	1,041	200	0.2028 (0.1194–0.2863)	93.5	108.6	<.0001
Malignancy/Cancer	32	6,986	197	0.0278 (0.0187–0.0383)	75.5	126.7	<.0001
Digestive system disease/GI disease	7	1,661	82	0.0504 (0.0267–0.0740)	82.2	33.7	<.0001
Immunity system‐related diseases							
Immunosuppression	3	604	13	0.0172 (0.0069–0.0276)	28.5	2.8	.2467
Immunodeficiency	3	1,612	11	0.0101 (0.000–0.0227)	71.5	7.0	.0297
Autoimmune diseases	3	425	4	0.0083 (0.0000–0.0169)	0.0	0.5	.7860
Infectious diseases							
Hepatitis B	5	1,801	40	0.0183 (0.0064–0.0303)	62.5	10.7	.0306
HIV	3	567	4	0.0058 (0.0000–0.0237)	64.6	5.6	.0590
Bacterial co‐infections/Bacteremia	2	675	8	0.0092 (0.0020–0.0164)	–	–	–
Chronic renal disease	21	5,659	119	0.0204 (0.0143–0.0266)	60.1	50.1	.0002
Chronic liver disease	16	3,254	92	0.0218 (0.0136–0.0314)	49.1	29.5	.0140
Chronic Respiratory disease/Pulmonary disease	15	3,215	150	0.0428 (0.0270–0.0586)	82.7	80.7	<.0001
Endocrinology disorder	8	1,338	130	0.0897 (0.0744–0.1049)	43.3	12.3	.0896
Hyperlipidemia	2	70	3	0.0197 (0.0000–0.0519)	–	–	–
Urinary system disease	2	781	23	0.0280 (0.0165–0.0396)	–	–	–
Hypertension	40	8,106	1,697	0.2354 (0.1914–0.2794)	96.5	1,127.2	<.0001
Diabetes	40	8,045	840	0.1168 (0.0980–0.1357)	87.0	300.4	<.0001
COPD^a^	23	5,610	148	0.0262 (0.0185–0.0339)	82.9	129.1	<.0001
Smoking	19	4,407	371	0.0827 (0.0586–0.1069)	87.9	149.4	<.0001

^a^ Chronic obstructive pulmonary disease.

## 4 DISCUSSION

Recently, the world has encountered an emergent outbreak posed by the novel coronavirus 2019, officially known as COVID‐19. This infection has become a global threat, endangering millions of lives worldwide. Hence, many experts, researchers, scientists, and clinicians are attempting to investigate various aspects of this new infection to find useful solutions for coping with COVID‐19. One of the various aspects of COVID‐19 is its impact on the CNS, as reported in a growing number of studies (Baig, 2020). In addition to the common symptoms in COVID‐19, several CNS symptoms such as headache and impaired consciousness have been observed in infected patients (Mao et al., 2020).

While most investigated the respiratory symptoms of COVID‐19, Mao et al. specifically examined the prevalence of neurological manifestations ranging from CNS to peripheral nervous system (PNS) and neuromuscular symptoms in an observational study on COVID‐19 patients. They demonstrated CNS presentations ranging from dizziness and headache to impaired consciousness, acute cerebrovascular disease, ataxia, and seizure (Mao et al., 2020). Based on the possible neuroinvasive potential of COVID‐19, in this systematic review and meta‐analysis, we analyzed those evidence indicating the involvement of CNS. We assessed 11,687 COVID‐19 adult patients from six countries. We reported that COVID‐19 patients commonly showed CNS symptoms, including headache, dizziness, and impaired consciousness. Headache (8.69%) was the most common CNS symptoms, followed by dizziness (5.94%) and impaired consciousness (1.9%). Exact reasons for headache, commonly seen in patients, remained unexplained. However, it can be due to COVID‐19‐related stress and anxiety (Garg, 2020). It is reported that the headache may also be related to the elevated level of inflammatory mediators and reduced cerebral blood flow in response to hypoxia (Jasti et al., 2020), but further studies are needed.

There are two main routes of CNS entry of COVID‐19 (hematogenous and peripheral nerves route) leading to CNS infection. In the hematogenous route, the virus infecting respiratory tracts can reach the CNS through the bloodstream via overcoming a strict obstacle known as the blood–brain barrier (BBB) (Desforges et al., 2014, 2020; Román et al., 2020; Sepehrinezhad et al., 2020; Swanson and McGavern, 2015). They also may enter the CNS through circumventricular organs, those CNS organs lacking the BBB (Chigr et al., 2020). The second route, a peripheral nerve, can provide the virus with a retrograde route in to access the CNS via an axonal transport machinery (Baig et al., 2020; Desforges et al., 2014, 2020; Román et al., 2020; Sepehrinezhad et al., 2020; Swanson and McGavern, 2015). In accordance with this finding, some previous studies on other types of coronaviruses indicate that coronaviruses can reach the brain via cranial nerves (e.g., olfactory, trigeminal nerve terminals in the nasal cavity) (Desforges et al., 2020; Li et al., 2016; Natoli et al., 2020; Netland et al., 2008).

Furthermore, SARS‐CoV‐2 can have indirect effects on the CNS (Zhou, Kang, et al., 2020). Cytokine storm as an immune system response during COVID‐19 infection could lead to the breakdown of the blood–brain barrier (BBB) (Liguori et al., 2020; Poyiadji et al., 2020). Infection of airway tissues by COVID‐19 in severe cases leads to impaired gas exchange, subsequently causing CNS hypoxia resulting in neural dysfunction (Abboud et al., 2020). More precisely, both cytokine storm and hypoxia which are frequently present in the severe condition of infection can contribute to making the BBB more permeable to the virus (Kaur & Ling, 2008; Zhou, Kang, et al., 2020).

There exists a wealth of evidence that supports the expression and distribution of the ACE2, the receptor for SARS‐CoV‐2, in the CNS (Jiang et al., 2013; Kawajiri et al., 2009; Li, Li, et al., 2020; Xia & Lazartigues, 2008; Xia et al., 2011; Xu et al., 2011; Zubair et al., 2020). Hence, ACE2 may be a potential target of COVID‐19 upon the entrance into the CNS, triggering its effects on CNS tissue (Baig et al., 2020). The presence of the virus in the central nervous system is also supported by some evidence reporting COVID‐19 in the CSF of the infected cases (Moriguchi et al., 2020; Zhou, Zhang, et al., 2020).

In our meta‐analysis, the mortality rate of COVID‐19 cases with at least one CNS symptom was 10.47%, which is much higher than the mortality rate of the general infected population (Borges do Nascimento et al., 2020). Such a mortality rate can indicate the importance of careful monitoring of CNS manifestations in COVID‐19 patients. This may be due to the effect of COVID‐19 on the brain stem and suppression of the cardiorespiratory control centers causing respiratory failure and death (Li, Bai, et al., 2020).

Moreover, recent studies have shown that COVID‐19 can accelerate the formation of the blood clot in the blood vessels, increasing the risk of cerebrovascular diseases in COVID‐19 patients (Choi et al., 2020; Hess et al., 2020). Hence, because the brain is nourished by a network of blood vessels, this could be indicative of the importance of cerebral vasculature investigations on the CNS symptoms in the COVID‐19 infection.

In a nutshell, attention to the CNS aspects of COVID‐19 infection has outstanding benefits for clinician's understanding of a very serious complication of this infection. At this point in time, researchers have mainly focused on finding medicinal treatments for respiratory symptoms of COVID‐19. However, it is necessary to investigate the various CNS manifestations of COVID‐19 since they are associated with increased severity and mortality (Mao et al., 2020). Not only respiratory system dysfunction, but also impairment of respiratory control centers in the CNS (brain stem) can induce acute respiratory failure (Carvalho et al., 2011; Li, Bai, et al., 2020). Therefore, considering all effective factors, it can provide clinicians to choose the best way in an attempt to manage this pandemic more efficiently.

## 5 LIMITATIONS

There are several limitations in our systematic review and meta‐analysis. Since in this ongoing pandemic, most of the investigations have conducted on typical signs and symptoms of COVID‐19. Thus, the number of studies on the atypical complications of COVID‐19, such as CNS presentations, is partially low. Moreover, there exist many COVID‐19 preprint papers that have not yet undergone peer review. Additionally, five studies included in our meta‐analysis reported headache and/or dizziness as one symptom in COVID‐19 cases. Because we were not sure that headache and/or dizziness is resulted from headache or is a consequence of the dizziness, it would be challenging to categorize headache and/or dizziness in the subgroup of dizziness or headache. Hence, in our meta‐analysis, it was not reported as a CNS manifestation and is implied as a separate symptom (Table 3).

## 6 CONCLUSION

COVID‐19 is a global problem that currently affects millions of people. This highly pathogenic virus can affect various parts of the human body. Although the respiratory tract has been mainly targeted by COVID‐19, the central nervous system can be affected significantly. In addition, patients with more severe illness showed more CNS symptoms, which may bring on worsen clinical conditions. This study achieved an important estimation for the incidence of neurological manifestations in patients with COVID‐19. The results of our survey may be helpful for clinicians for better diagnosis and management of CNS signs and symptoms in patients with COVID‐19.

## 7 AUTHOR CONTRIBUTORS

SH.N. and S.D. conceptualized and designed the study; A.A.J., S.M., S.S., S.S., and M.H. involved in acquisition of data; S.D. analyzed and interpreted the data; S.H.N., S.D., A.A.J. S.M., S.S., and S.M.P. drafted the manuscript; SH.N., S.D., A.A.J., F.A., H.E., and D.F. critically revised the article.

## CONFLICT OF INTEREST

None.

### Peer Review

The peer review history for this article is available at https://publons.com/publon/10.1002/brb3.2025.

## Data Availability

The data that support the findings of this study are openly available.
